# Temperature versus Relative Humidity: Which Is More Important for Indoor Mold Prevention?

**DOI:** 10.3390/jof8070696

**Published:** 2022-06-30

**Authors:** Haoxiang Wu, Jonathan Woon Chung Wong

**Affiliations:** Department of Biology and Institute of Bioresource and Agriculture, Hong Kong Baptist University, Hong Kong, China; kubeng@life.hkbu.edu.hk

**Keywords:** temperature, relative humidity, wet-dry cycles, indoor mold prevention

## Abstract

Temperature is known as one of the abiotic factors that can affect mold growth. Many mold growth prediction models consider temperature as one of the parameters that can significantly impact mold growth indoors, and hence temperature has been targeted by different indoor mold prevention strategies on different premises. For example, European guidelines for libraries suggest a temperature of 19 °C to preserve books. However, running low temperature air-conditioning (AC) costs substantially more energy, and thus a higher temperature (e.g., 25.5 °C) has been regularly proposed as the recommended indoor temperature for general indoor environments in Hong Kong. It is, therefore, needed to understand whether or not the reduction of indoor temperature would lead to better effectiveness of mold prevention. Using *Cladosporium cladosporioides* (*C. cladosporioides*) as the model, its germinating spores were challenged in *C. cladosporioides* to wet-dry cycles with different combinations of relative humidity (RH, 40%, 60% and 80%) and temperature (19 °C and 28 °C) levels. The survival, lipid peroxidation and catalase (CAT) activity of the treated spores were monitored and compared. *C. cladosporioides* spores showed similar levels of viability, lipid peroxidation and CAT activity when they were exposed to 19 °C and 28 °C at the same RH, but substantially lower survival and higher oxidative stress were observed under the wet-dry cycles with 40% RH dry periods compared with 60% and 80% RH at both temperatures, suggesting that indoor temperature does not tend to affect the resistance of *C. cladosporioides* to wet-dry cycles as significantly as the RH level of the dry period. Collectively, this study suggests a more important role for moisture over temperature in indoor mold prevention. The outcome of this study may facilitate the sustainable management of indoor mold problems in buildings.

## 1. Introduction

Due to the adverse health effects brought by molds, building scientists have developed mold growth models to predict mold development under different environmental conditions. As indicated by the growth models, temperature is one of the abiotic factors proven to significantly affect mold growth [1]. Therefore, temperature has been one of the targets for mold control in indoor environments [2].

As one of the important factors affecting mold growth, the temperature effect has been widely investigated by researchers. The investigations of the temperature effect were initiated under a constant state, which focused on the impacts on indoor mold growth brought by different temperature levels [1,3,4]. Moreover, the production of mycotoxins was also found to be significantly affected by temperature [5], highlighting the need for considering temperature as one of the factors that would not only affect mold growth but also potentially compromise human health if it is not properly maintained. Later, upon realizing that constant states of temperature are artificial environments in laboratories rather than real-world situations, researchers began to place more focus on the effects of cyclic temperature levels on the growth of indoor molds. Johannson et al. (2013) exposed mold spores to transient temperature conditions and drew the conclusion that dynamic temperature appeared to slow down mold growth compared with steady-state temperature levels [6]. These previous studies tend to support the important role of temperature in influencing the responses of molds.

European guidelines suggest a temperature range of 19–24 °C for preserving paper products. In particular, for premises where important materials are stored; e.g., libraries, the temperature is usually maintained at relatively low levels (e.g., at around 19 °C) to slow down mold activities, in order to protect paper materials from fungal deterioration [7]. As per the updated VTT model developed by Ojanen et al. (2010), molds take significantly longer to germinate at 19 °C compared with 25.5 °C on building materials that are sensitive to mold growth [1]. Therefore, maintaining a low temperature has been widely believed to be effective at preventing indoor mold contamination. However, the facility management team for university campuses on occasion receives complaints from students regarding the low temperature in the library. It is reported that a drop of 1 °C in the room temperature consumes 3% more energy [8], and simultaneously, the operation of ACs emits carbon dioxide (CO_2_) which would accelerate global warming. Therefore, in order to balance indoor mold hygiene and environmental sustainability, it is necessary to investigate if such a low temperature is needed to be maintained in particular venues.

The aim of this study is to investigate the effect of temperature on the resistance of indoor molds to wet-dry cycles, so as to understand the necessity of maintaining a costly low temperature indoor environment. In this study, using *Cladosporium cladosporioides* (*C. cladosporioides*) as the model, its germinating spores were exposed to wet-dry cycles with the combination of two temperatures (19 °C and 28 °C) and three relative humidity (RH) levels (40%, 60% and 80%). The two tested temperature levels, 19 °C and 28 °C represent the low temperature in libraries and unconditioned atmospheric temperature, respectively; meanwhile, 40% and 60% RH mimic the lowest and normal indoor humidity that air-conditioning units or dehumidifiers can maintain, and 80% is a common unconditioned atmospheric RH level. Afterward, the germination percentage, lipid peroxidation level and CAT activity of the treated molds were measured and compared. The results of this study help to facilitate the sustainable management of mold hygiene problems in buildings.

## 2. Materials and Methods

### 2.1. Tested Organisms and Growth Conditions

*C. cladosporioides* stain ATCC^®^ 16022™ was ordered from the American Type Culture Collection (ATCC; Manassas, Va, USA) and used in our experiments due to its common isolation from indoor environments and role as a model mold species for standard tests [9]. Mold cultures were routinely grown on malt extract agar (MEA; HKM, Guangzhou, China) plates at 28 °C for a week prior to the experiments.

### 2.2. Mold Survival under Moisture Dynamics

The experimental setup used in this study is similar to the one detailed previously [10,11]. Briefly, spores of *C. cladosporioides* were harvested, washed and enumerated according to the standardized method [12]. Afterward, 100 μL of spore suspension with a density of 1 × 10^6^ spores/mL was inoculated onto a cellulose membrane (47 mm diameter, 0.2 μm pore size) overlying a 0.99 water activity (a_w_, equivalent to 99% RH) MEA plate. Inoculated spores were spread out homogeneously using a disposable plastic spreader, then cultured in the isotropic swollen stage (confirmed by a light-microscope). Afterward, membranes with swollen spores were transferred to low a_w_ MEA plates (0.4 a_w_, 0.6 a_w_ or 0.8 a_w_) and incubated under 40%, 60%, or 80% RH for up to 15 days at either temperature (19 °C or 28 °C) in a digital hygrothermal incubator (Bluepard, Shanghai, China). The adjustment of the a_w_ levels of agar plates was achieved by supplementing glycerol [10]. Next, membranes with spores were re-exposed to wet conditions (0.99 a_w_, 28 °C) for up to one month to assess their viability. These wet-dry cycles are termed as moisture dynamics. Spores able to form a germ tube longer than or equal to their longest dimension after exposure to moisture dynamics were defined as viable. Germination percentage was used to present the viability of spores exposed to moisture dynamics, which was assessed by cutting 1 cm^2^ of the membrane and counting the percentage of germinated spores under a light-microscope during re-exposure to the wet condition.

### 2.3. Quantification of Oxidative Stress

Elevated oxidative stress leads to the generation of reactive oxygen species (ROS), and thus damages cell lipids. As a consequence of oxidative damage, the lipid peroxidation level of dried molds was measured with a Lipid Peroxidation (MDA) Assay Kit (Abcam) following the protocol provided by the manufacturer, which assesses the formation of malondialdehyde (MDA) in mold samples. Immediately upon the completion of wet-dry cycles, treated mold spores were moved into sterile ultrapure water immediately and subjected to homogenization. The determined MDA concentration in mold samples was then normalized with the protein concentration present in the samples, which was quantified using a Pierce^TM^ BCA Protein Assay Kit (Thermo Fisher Scientific, Waltham, MA, USA).

### 2.4. Characterisation of Antioxidant Responses

CAT is the key antioxidant enzyme produced to decompose H_2_O_2_ oxidative stress. As confirmed in our previous study, catalase (CAT) is the most significantly changed antioxidant enzyme in response to moisture dynamics [11]. Therefore, CAT activity was used to represent antioxidant responses in this study. The CAT activity of treated molds was quantified using a Catalase Assay Kit purchased from Cayman Chemical (Ann Arbor, MI, USA) following the protocol suggested by the manufacturer. Catalase activities of all samples were minimized with the protein concentration.

### 2.5. Statistical Analyses

The differences in the levels of lipid peroxidation and CAT activity in *C. cladosporioides* under different temperature and RH levels were compared using repeated measures of one-way analysis of variance (ANOVA) with Duncan’s post hoc test in SPSS v.24, in order to determine whether significant differences in oxidative stress and antioxidant response were found between the tested mold species. Differences between means with a *p*-value lower than 0.05 (*p* < 0.05) were regarded as statistically significant.

## 3. Results

### 3.1. Viability of C. cladosporioides under Moisture Dynamics

*C. cladosporioides* spores were cultured to the isotropic swollen stage prior to the exposure to different water conditions. The mean length of swollen spores measured in this study is 7.64 μm (± 0.18 μm SD), which is comparable to 7.51 ± 0.96 μm in Quintana-Obregón et al.’s (2011) study [13]. Over 80% of spores were at a synchronized developmental stage.

**Figure 1:**
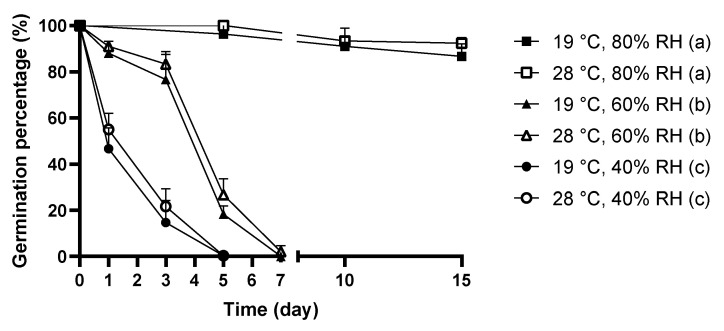
The germination percentage of *C. cladosporioides* spores exposed to moisture dynamics with different combinations of RH and temperature levels. The changes in germination percentage under moisture dynamics were analyzed by repeated measures one-way ANOVA. Grouping was performed by Duncan’s post-hoc test. Letters in parentheses indicate different groupings.

The germination percentage of the dehydrated mold spores was used as an indicator to assess mold viability. The viability of *C. cladosporioides* spores under moisture dynamics with different combinations of temperature and RH levels is presented in Figure 1.

The viability of *C. cladosporioides* decreased as the drying time extended at both 19 °C and 28 °C when they were exposed to wet-dry cycles with dry periods at 40% and 60% RH; meanwhile, there was not a significant decrease in viability when molds were incubated under 80% RH. Similarly, there was no significant difference found in the survival of *C. cladosporioides* between 19 °C and 28 °C when the RH was fixed, but 40% RH led to significantly lower viability in *C. cladosporioides* than 60% and 80% RH at both temperature levels.

The lowest survival was determined when *C. cladosporioides* was incubated under 40% RH dry periods. The viability of molds dropped sharply to 47% at 19 °C and 55% at 28 °C after 1 day, then to 15% at 19 °C and 22% at 28 °C upon 3-day-drying. After 5 days, all the spores dehydrated under 40% RH at 19 °C and 28 °C were inactivated and no resumption of growth could be observed followed by subsequent rewetting. When dry periods of 60% RH were adopted, for both temperature levels, the viability of *C. cladosporioides* declined to approximately 80% after the first 3 days. Then, the most dramatic reduction in the viability of *C. cladosporioides* was observed between the and fifth day, where the survival of mold spores dropped to 20%. All spores were inactivated when the dry periods were further extended to 7 days for 19 °C and 28 °C.

In contrast, dry periods of 80% RH at neither 19 °C nor 28 °C appeared to inactivate *C. cladosporioides* spores within 15 days. Similar survival was observed throughout the 15-day dry periods and almost all treated spores at 19 °C and 28 °C restored growth after a 15-day 80% RH period. Overall, *C. cladosporioides* spores subjected to wet-dry cycles showed similar levels of survival between 19 °C and 28 °C when the same RH level was maintained while significantly lower viability was observed when the RH was reduced to 40% compared with 60% and 80% at both temperatures.

### 3.2. Oxidative Stress Encountered in C. cladosporioides under Moisture Dynamics

**Figure 2:**
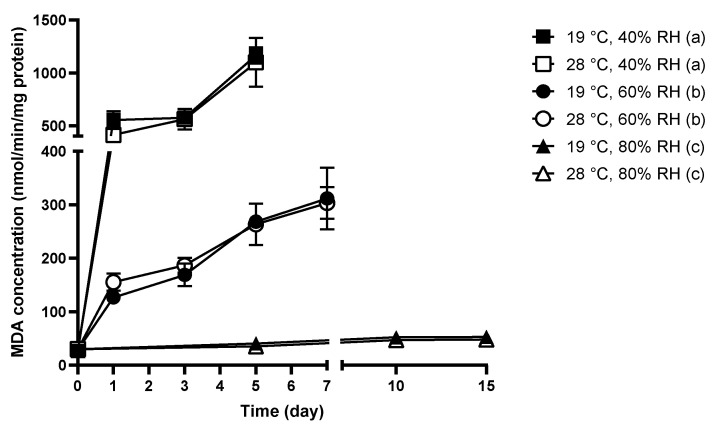
Lipid peroxidation level of *C. cladosporioides* spores exposed to moisture dynamics with different combinations of RH and temperature levels. The changes in MDA concentration under moisture dynamics were analyzed by repeated measures one-way-ANOVA. Grouping was performed by Duncan’s post-hoc test. Letters in parentheses indicate different groupings.

Lipid peroxidation has been demonstrated to give a clear indication of the oxidative damage encountered by molds; thus, the formation of MDA, which is used to reflect lipid peroxidation level, was monitored in *C. cladosporioides* spores in the present study. Results are shown in Figure 2.

Under wet conditions (99% RH, 0 days), *C. cladosporioides* formed a low background level of MDA concentration, which was approximately 30 nmol/min/mg protein. A substantial increase in MDA concentration was measured when 40% or 60% dry periods were introduced at 19 °C and 28 °C. General climbing trends in MDA concentration were observed for dry periods of 40% and 60% RH at both tested temperatures.

The highest MDA formation was detected in *C. cladosporioides* incubated under 40% RH dry periods. In a one-day 40% RH period, MDA concentration increased to 411 nmol/min/mg protein at 19 °C and 553 nmol/min/mg protein at 28 °C. MDA concentration reached 1100 nmol/min/mg protein at 19 °C and almost 1168 nmol/min/mg protein at 28 °C after the fifth day. For both temperatures at the 60% RH, the concentration of MDA in *C. cladosporioides* was approximately 130 nmol/min/mg protein upon the first day of drying and gradually ascended to over 300 nmol/min/mg protein after 7 days.

With respect to *C. cladosporioides* spores incubated at 80% RH, the lipid peroxidation level at both tested temperatures tended to be much lower than at 40% and 60% RH. A flattening change in MDA concentration (around 40 nmol/min/mg protein) was measured for both 19 °C and 28 °C, which was similar to the control wet condition (99% RH). Generally, the formation of MDA in *C. cladosporioides* spores was similar between 19 °C and 28 °C, when the RH was fixed, but a markedly higher MDA concentration was determined in *C. cladosporioides* spores exposed to lower RH levels.

### 3.3. CAT Activity of C. cladosporioides under Moisture Dynamics

**Figure 3:**
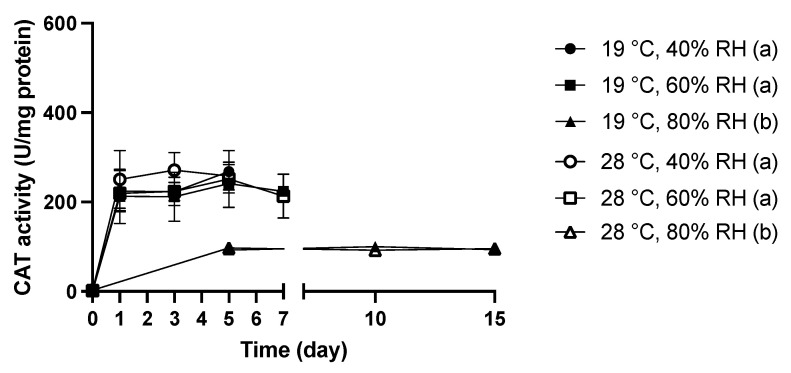
CAT activity of *C. cladosporioides* spores under wet-dry cycles with different combinations of RH and temperature. The changes in CAT concentration under moisture dynamics were analyzed by repeated measures one-way ANOVA. Grouping was performed by Duncan’s post-hoc test. Letters in parentheses indicate different groupings.

As an important antioxidant enzyme produced to detoxify oxidative stress, CAT activity was monitored in *C. cladosporioides* under wet-dry cycles and is shown in Figure 3.

Under control conditions (0 day), very low (2 U/ mg protein) CAT activity was found in *C. cladosporioides*, and all tested combinations of wet-dry cycles considerably induced CAT activity in *C. cladosporioides*.

When dry periods of 40% and 60% RH at 19 °C and 28 °C were introduced, the CAT activity of *C. cladosporioides* increased to around 260 U/mg for all of the four tested combinations and remained insignificantly different during the incubation under dry periods. At an RH of 80%, there were significant increases in CAT activities when compared with the background level, but the level of CAT activities detected in *C. cladosporioides* was approximately 95 U/mg protein for both temperatures, which was markedly lower than that measured under 40% and 60% RH. Generally, the CAT activities achieved by *C. cladosporioides* under 40% and 60% RH at 19 °C and 28 °C were similar and found to be higher than the CAT activities detected under the wet-dry cycles with a RH of 80% RH (19 °C, 80% RH and 28 °C, 80% RH).

## 4. Discussion

Extensive studies have demonstrated that molds accelerate their growth rate when the temperature approaches the optimum level [4,14], and thus, all mold growth prediction models take temperature into account when the growth of molds needs to be predicted [1,4]. However, temperature is also an important factor influencing occupants’ comfort, and therefore, should be kept within a certain range; an “Excellent” level of indoor air quality (IAQ) requires an indoor temperature between 20 °C and 25.5 °C. In Hong Kong, in light of sustainability, 25.5 °C has been regularly proposed to be a recommended temperature for air-conditioning (AC) systems. As one of the special cases, low temperature is usually maintained in libraries for the sake of book preservation. The low temperature of 19 °C is suggested by European guidelines to preserve books, which could cost nearly 20% more energy compared with 25.5 °C. Determining whether or not it is necessary to run such an energy-consuming mold prevention strategy is useful. The findings of this study may help in developing a more sustainable and reasonable AC management regime for mold prevention in indoor environments, especially libraries.

### 4.1. Insignificantly Different Resistance to Wet-Dry Cycles between 19 °C and 28 °C

As shown in Figure 1 and Figure 2, *C. cladosporioides* spores did not show significantly different tolerance toward wet-dry cycles between 19 °C and 28 °C, as reflected in the similar viability and lipid peroxidation levels. The temperatures of 19 °C and 28 °C represent typical low and unconditioned indoor levels, respectively. The insignificantly different tolerance towards wet-dry cycles revealed in this study implies that indoor temperature does not tend to significantly affect the effectiveness of mold prevention, suggesting that temperature control is not an effective approach and it may not be necessary to maintain a low temperature in indoor environments.

Although this is the first study to investigate the effects of temperature on the resistance of indoor molds to wet-dry cycles, making the results not directly comparable to other work, the insignificant role of indoor temperature revealed in this study meets the expectations of Aihara et al. (2002) and other mold growth models [4,14]. It is reported that *C. cladosporioides* showed similar resistance to suboptimal moisture conditions within the range of 19 °C to 28 °C. Moreover, the isopleth model also supports the finding that mold growth rate is similar at 19 °C and 28 °C for all tested RH levels.

Notably, the above-referenced studies that examined temperature effects were conducted under constant moisture conditions, and the influence of indoor temperature on the tolerance of indoor molds towards wet-dry cycles has not been explored. Some mold growth models (e.g., the VTT model) indicate a significantly slower growth at 19 °C compared with 28 °C, which may over-emphasize the necessity for maintaining a low indoor temperature [1]. In reality, the water supply for molds in indoor environments can fluctuate substantially because of occupants’ activities, such as cooking and showering, and therefore, the temperature effect revealed under constant water conditions is not sufficient for understanding its role in real-world building contexts. The findings of our current study hint that the significance of the temperature effect tends to be less than expected and it is more important to run a wet-dry cycle regime with low RH periods for the purpose of indoor mold prevention.

### 4.2. Relative Humidity of Dry Periods Is More Critical Than Temperature for Mold Prevention in Indoor Environments

According to Figure 1 and Figure 2, when the relative humidity was fixed at 60% RH, *C. cladosporioides* spores did not exhibit markedly different viability and lipid peroxidation levels between 19 °C and 28 °C, suggesting that *C. cladosporioides* encountered similar oxidative stress at these temperature levels under wet-dry cycles. However, when the temperature was maintained at either 19 °C or 28 °C, *C. cladosporioides* displayed substantially lower survival and higher lipid peroxidation levels at 40% RH compared with 60% and 80% RH. These results suggest that within the indoor context, the importance of the RH in dry periods tends to be higher than the temperature, strengthening the observation that RH is a more important factor in determining mold survival when compared to temperature in the indoor environment.

Although no researcher has compared the impacts brought by temperature to RH on the tolerance of indoor molds to wet-dry cycles, the expectation that the degree of water stress (i.e., RH) is more significant than the temperature suggested by this study is consistent with others’ work. It was reported by Aihara et al. (2002) that a reduction of 0.01 a_w_ (i.e., 1% RH) appeared to significantly delay the growth of *C. cladosporioides* and *C. sphaerospermum*, whereas a decrease of 6 °C was required to acquire the same growth delay. Briceño and Latorre (2008) also observed a higher growth rate at 0.98 a_w_ than 0.96 a_w_ (equivalent to the difference of 2% RH) in *C. cladosporioides* and *Cladosporium herbarum* [15]. In addition, Krus et al. (2007) showed that only when the temperature was 10 °C apart from the optimum level, a significant difference could be observed, while a 5% lower RH was able to yield an observable slower growth rate [16]. The results of these studies help support the insignificant differences in survival observed in the current work.

It is worth mentioning that although 40% RH was demonstrated to cause lower viability and severe oxidative stress in *C. cladosporioides*, this knowledge does not necessarily bind a lower RH to a more detrimental consequence or higher stress level. Wyatt et al. (2015) observed a better survival of two fungi, *Talaromyces macrosporus* and *Neosartorya fischeri*, under stringently dried conditions (0% RH) compared with air dried at 40–60% RH [17]. Segers et al. (2016) believed that this was because of the low mobility of molecules at 0% RH, which suppressed the detrimental chemical reactions [12]. Therefore, some extreme conditions may to some extent act as a protective effect against ambient stresses, and thus, it is not a definite rule that lower a_w_ or RH will contribute to a more stressful environment.

### 4.3. Similar CAT Activity Determined at 19 and 28 °C May Explain the Insignificant Effect of Temperature under Moisture Dynamics

Temperature is known to impact the metabolic activity of microorganisms by affecting their enzyme activities. Enzymes peak their activities at optimum working temperature, and therefore, the suboptimal temperature would reduce the protective effect of enzymes when microbes encounter unfavorable environments. Tang et al. (2007) agreed that shifts in ambient temperature could reduce the survival of airborne bacteria because the changing temperature affects enzyme activities and consequently impacts the metabolic activity and viability of microbes [18].

Indoor molds typically peak their growth between 25 °C and 30 °C, which implies that the enzymes of the majority of indoor molds may exhibit an optimum working efficiency within this temperature range. As shown in Figure 2, except for the case of 80% RH, CAT activities carried by *C. cladosporioides* under a RH of 40% or 60% RH as well as a temperature of 19 °C or 28 °C were similar. Although 40% RH caused much higher oxidative stress and lower survival, the CAT activities measured, resembled those under an RH of 60%, suggesting that dry periods of 60% RH have already induced the maximum capacity of the CAT enzyme, and hence even when a more stressful RH level (40% RH) was introduced, the defense (CAT activity) still remained similar. The unobservable difference in the CAT activity alongside the similar oxidative stress encountered determined in Figure 2 and Figure 3 may help explain the insignificant effect of temperature on the resistance of *C. cladosporioides* to wet-dry cycles—when the stress and defense level remain insignificantly different, the consequences (viability, representing resistance to wet-dry cycles) would also likely be similar. On the other hand, since 60% RH has already induced the maximum capacity of the CAT enzyme in *C. Cladosporioides*, when the RH was reduced to 40% and severer stress was imposed, lower viability was expected. Therefore, it also makes sense that 40% RH yielded lower viability in *C. cladosporioides* compared with 60% and 80% RH.

### 4.4. Implications of the Study

Libraries normally store numerous special collections, and hence the management team would choose to minimize the risk of fungal contamination. According to some mold growth models, e.g., the updated VTT model, a lower temperature can further slowdown mold activities, and consequently, a low temperature is usually maintained in libraries. From time to time, complaints regarding the low temperature in libraries have been experienced. Many management teams have published announcements and explanations on the low temperature maintained in libraries. As a large part of operation costs, the AC accounts for over 30% of building maintenance charges [8]. Furthermore, several libraries operate continuous AC to minimize the risk of mold outbreaks [7], which would lead to an even higher cost if the low temperature is also maintained.

However, the results of this study stress the necessity of running ACs in a constant low temperature mode as long as the humidity is properly maintained. Figure 1 and Figure 2 indicate insignificant differences in survival and oxidative stress encountered between 19 °C and 28 °C when *C. cladosporioides* spores were exposed to wet-dry cycles; while dry periods at a lower RH rendered markedly lower mold viability and higher encountered oxidative stress, which implies that the reduction of indoor humidity would probably contribute to a better mold prevention effectiveness than running ACs at cold temperature.

In addition, two commonly found indoor molds, *C. cladosporioides* and *Cladosporium halotolerans*, are known to be psychrotolerant [9]. They have been isolated from cold environments, such as fridges and arctic regions, respectively, which may be a sign that these mold species are able to maintain proper enzymatic activity even when the environment is unfavorably cold. The lowest acceptable temperature is ascertained to be 19 °C, and the insignificant effect imposed by 19 °C on mold survival under wet-dry cycles compared with 28 °C revealed in this study may be extrapolated to other common indoor molds, such as *C. halotolerans*, which further challenges the effectiveness of temperature control.

Given that indoor temperature does not tend to be as critical, it may be feasible to use dehumidifiers instead of ACs to achieve moisture control in order to save energy. The use of dehumidifiers to replace ACs is not limited to libraries and could also be applied to other indoor environments, such as offices and residential areas. In a typical 20 m^2^ office, a 280 Watts-hour dehumidifier only accounts for 34% of the costs caused by AC operation (using 1.5-ton AC as an example). Hence, the facility management team may consider operating ACs at a higher temperature (e.g., 25 °C during office hours for occupants and temperature levels that do not require cooling after office hours) or using dehumidifiers instead to balance sustainability and mold hygiene.

## 5. Conclusions

In this study, *C. cladosporioides* spores were challenged in wet-dry cycles with different combinations of RH (40%, 60% and 80%) and temperature levels (19 °C and 28 °C). We present insignificant differences in the viability, lipid peroxidation levels and CAT activity of *C. cladosporioides* between the two tested temperature levels under wet-dry cycles. When the temperature was fixed at either 19 °C or 28 °C, markedly higher oxidative stress and lower viability in *C. cladosporioides* spores were found at 40% RH compared with 60% and 80% RH. The results stress the necessity of maintaining a low temperature in indoor environments, such as libraries and imply that moisture control tends to be more crucial than maintaining a cold environment for the sake of indoor mold prevention.

## Data Availability

Data are contained within the article.
